# Analysis of Social Effects on Employment Promotion Policies for College Graduates Based on Data Mining for Online Use Review in China during the COVID-19 Pandemic

**DOI:** 10.3390/healthcare9070846

**Published:** 2021-07-05

**Authors:** Tinggui Chen, Jingtao Rong, Lijuan Peng, Jianjun Yang, Guodong Cong, Jing Fang

**Affiliations:** 1 School of Statistics and Mathematics, Zhejiang Gongshang University, Hangzhou 310018, China; rjt323@126.com (J.R.); Cherrylijuanpeng@163.com (L.P.); 2 Department of Computer Science and Information Systems, University of North Georgia, Oakwood, GA 30566, USA; Jianjun.Yang@ung.edu; 3 School of Tourism and Urban-Rural Planning, Zhejiang Gongshang University, Hangzhou 310018, China; cgd@mail.zjgsu.edu.cn; 4 Department of Social Sciences, Zhejiang Gongshang University, Hangzhou 310018, China; fjhust@mail.zjgsu.edu.cn

**Keywords:** COVID-19, data mining, college graduates, employment policy, policy evaluation

## Abstract

As an important part of human resources, college graduates are the most vigorous, energetic, and creative group in society. The employment of college graduates is not only related to the vital interests of graduates themselves and the general public, but also related to the sustainable and healthy development of higher education and the country’s prosperity through science and education. However, the outbreak of COVID-19 at the end of 2019 has left China’s domestic labor and employment market in severe condition, which has a significant impact on the employment of college graduates. Based on the situation, the Chinese government has formulated a series of employment promotion policies for college graduates in accordance with local conditions to solve the current difficulties in employment of college graduates during the COVID-19Pandemic. Do these policies meet the expectations of the people? Is the policy implementation process reasonable? All these issues need to be tested and clarified urgently. This paper takes the employment promotion policy of college graduates under the COVID-19 as the research object, uses the PMC index model to screen the policy texts, obtains two perfect policy texts, and uses the Weibo comments to construct the evaluation model of policy measures support degree to analyze the social effects of employment promotion policies for college graduates. The results show that the public’s support degree with the employment promotion policies for college graduates under COVID-19 needs to be improved. Among them, the public has a neutral attitude towards position measures and transference measures but is obviously dissatisfied with subsidy measures and channel measures. Finally, suggestions for improving policy are given to make the employment policy in line with public opinion and effectively relieve the job hunting pressure of college graduates.

## 1. Introduction

The COVID-19 pandemic has plunged the global economy into panic and trouble [1,2]. It has caused a large-scale shutdown of work and production in the whole society, which has impacted social and economic development and the overall employment environment, and has seriously affected the employment of college graduates. As employment is the foundation of people’s livelihood, steadying employment stabilizes the economy and people’s expectations, livelihood, and confidence. College graduates are the main force in the job market, so promoting their stable and smooth employment is an important part of the harmonious development of the current society and the stable operation of the economy. In response to the impact of COVID-19 on the employment of college graduates, the Chinese government has launched a series of employment stabilization measures: the Ministry of Education launched a 24.365 full-day online campus employment service platform, extended the time limit for college graduates to register and settle down, expanded the enrollment scale of students in Master programs andthe students upgraded from associate degree to baccalaureate, etc. In addition, if the unemployment of college graduates is not properly resolved, a waste of talents and social instability will take place. Social stability is a prerequisite for economic development, and social turbulence will inevitably affect the orderly development of the economy. For the above reasons, scientific evaluation of employment promotion policies for college graduates under the COVID-19 and corresponding suggestions are of important theoretical and practical significance.

At present, the research on the employment promotion policy of college graduates mostly focuses on the analysis of employment problems and employment-related promotion policies from a macro perspective. In particular, it mainly focuses on theoretical research and the evaluation for the effect of policy implementation. For example, Zhu and Chen [3] made comments on the development and reform of the employment-related promotion policy for college students after the reform of China’s economic system and put forward suggestions for improving the employment promotion policy. Ercument and Emine [4] conducted the employment evaluation experiment of the students majoring in architecture in Hong Kong and Shanghai, and found that improving students’ ability, strengthening specialization, and guiding practical ability can effectively promote the comprehensive ability of graduates in group work. However, there are few literatures from the perspective of policy-making to comprehensively evaluate different policy texts, summarize key policy content from them, and use relevant comments to analyze the social effects of the policy. In view of the inadequacy of existing research, this paper uses the Policy Modeling Consistency (PMC) index model to rate the employment promotion policy for college graduates, screens important policy content, collects Weibo comments on related topics, and constructs a support evaluation model for policy measures to respond to college graduates under the COVID-19. Further, thepaper analyzes the social implementation effects of the employment promotion policy, and finally makes recommendations based on the results of the analysis.

The structure of the paper is as follows. Section 2 is a literature review. Section 3 uses the PMC index model to extract key content in the employment promotion policy for college graduates under the COVID-19. Section 4 uses the policy support degree evaluation model to analyze the impact of policy support degree evaluation model on employment promotion policy. Section 5 is the conclusions and the future work prospects.

## 2. Literature Review

The current research on employment policy mainly focuses on the following two aspects: one is the analysis of the factors affecting employment policy. The other is evaluating the effect of employment policy implementation.

Regarding the research on the factors affecting employment policies, some scholars have carried out research on individual employability, and believed that internal motivation [5], superior expectations [6] and organizational environment [7] are the main factors affecting individual employment innovation ability. The typical literature is as follows: A study conducted by Genco et al. [8] at the University of Massachusetts showed that freshmen with working experience performed better when given incentives and technical flexibility than general graduates, who have obvious differences in incentives and flexibility. Xu et al. [9] proposed that alumni feedback information systems, social evaluation systems, etc. are important resources for improving the quality of talent training in colleges and universities and for guiding and adjusting employment policies. Zhang et al. [10] and Yu [11] used questionnaire survey methods to analyze the employment area selection and graduate employment rate of college graduates in different regions. Yang and Yang [12] used regression analysis in mathematical statistics based on a large-scale sample survey of employment status of college graduates across the country, and studied the factors that affect the employment competitiveness of college graduates, including school reputation and status, employment service information, academic qualifications, academic work, and work ability, employment expectations, etc. Li and Lin [13] analyzed the factors that promoted graduates’ employment and discussed their relations between each other using general system structure theory, and they constructed an employment promotion system model and its structure. Zhang [14] used a Bayesian data mining classification algorithm to explore the employment option of college graduates. Through training the existing data of the employment option of college graduates, he analyzed the feedback of graduates that were satisfied with their jobs. Furthermore, he gained the classification feature set rules and established a classification model about the employment option of college graduates. It was proved by the experiments that this model was selected user groups with high accuracy. Based on the survey data of the graduates of Jiangsu university for five consecutive years, Jie [15] adopted the method of gray relational analysis to conduct an empirical study on factors affecting the employment of college students. The results showed that there were 15 main factors affecting the employment prospects of college students. Among them, the importance of the ability of active learning was the most important one, followed by working ability. From the government, universities, enterprises, society, graduates, and other subjects, Xi and He [16] analyzed the role of a series of employment promotion and entrepreneurship guidance policies put forward by a provincial government. The results showed that the policy had not achieved the expected effect in promoting college students’ independent entrepreneurship. Furthermore, the propaganda strength of the policy, the pertinence of the support object, the effectiveness of the policy, and the effectiveness of the policy supporting services for entrepreneurship need to be further improved. According to the above literature analysis, most of the research on employment policies for college graduates uses qualitative methods to explore the influencing factors of employment policies, and few policies start from the policy formulation itself to make a scientific evaluation of the social effects after the policy is implemented.

The research on the evaluation of the implementation effect of employment policies began with Edward A. Suchman’s five-category evaluation, followed by Oville F. Poland’s “Three E” evaluation classification framework and Wollmann’s classic policy evaluation [17]. Yet most of the literature is based on empirical research, using case analysis, field questionnaire surveys, and other methods. Some examples of typical documents are as follows: Song [18] takes Guangdong Province and puts forward suggestions for optimizing policies. Hu and Chen [19] first took 1500 college students in Henan Province as the survey object and conducted a data survey on all the employment promotion policies of college students in China. If the employment policy is very practical and highly recognized by college students, it is followed by satisfaction with the employment services provided by the government. Zhao and Yao [20] jointly established the CR2 model and the corresponding projection model and constructed an evaluation index system. Using the data envelopment analysis method, they empirically analyzed the effectiveness of the public employment service policies for college students in China from 1999 to 2006. Zhang [21] believes that the historical evolution of the employment policy for college graduates has experienced three stages: the planned economic system period, the educational system reform and development period, and the social entity market economy establishment and development period. Each stage has its own different characteristics. Its characteristics are used to solve the employment problem of college graduates. The literature shows that most of the research is conducted on specific objects to test the effect of the policy after the release, and the research objects of this method are relatively one-sided and have certain timeliness. In turn, such research can neither dynamically grasp the audience’s views on the policy in real time, nor make optimization suggestions for policy formulation based on the public opinion response of the people.

To sum up, the current academic circles have carried out research on employment policies that rarely combine public opinion with the evaluation and analysis of employment policies. Therefore, this paper starts from the policy text itself, ranks policy documents, digs out important policy content, trawls Weibo comments on this basis, and uses the comments to construct the employment promotion policy for college graduates under the COVID-19 to analyze the social effects of policy implementation.

## 3. Research Framework

**Figure 1:**
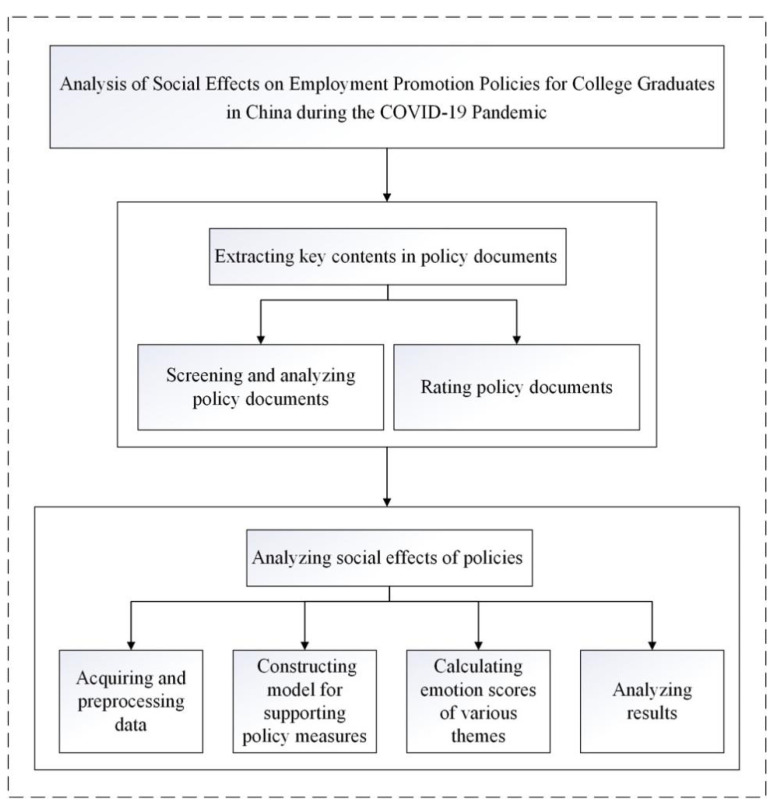
Research framework.

First, this paper collected employment promotion policy documents for college graduates released by the Chinese government during the COVID-19, conducted word frequency analysis, and rated the collected policy by PMC index model to select and summarize important policy measures. Then, related topics of 4 kinds of policy measures were searched, Weibo comments were trawled, and an evaluation model of support degree of policy measures was constructed to evaluate and analyze the public support degree of policy measures, so as to study the social implementation effect of employment promotion policy for college graduates. The framework of the paper is shown in Figure 1.

## 4. Extracting Key Contents of Employment Promotion Policy for College Graduates

### 4.1. Selecting and Analyzing Employment Promotion Policy

In order to extract the key contents of employment promotion policy for college graduates, this paper selects the related policy issued by the Chinese government from January 2020 to July 2020 during COVID-19, with reference to the graduation time of undergraduate and graduate students in previous years. In the acquisition process, following the principles of authority, rigor, completeness, and accuracy, the authors collected 16 typical policy documents that have important effects on the employment of college graduates from the websites of the Ministry of Human Resources and Social Security, People’s Republic of China (PRC), the Ministry of Education, the Central People’s Government, and other authority websites, as shown in Table 1.

**Figure 2:**
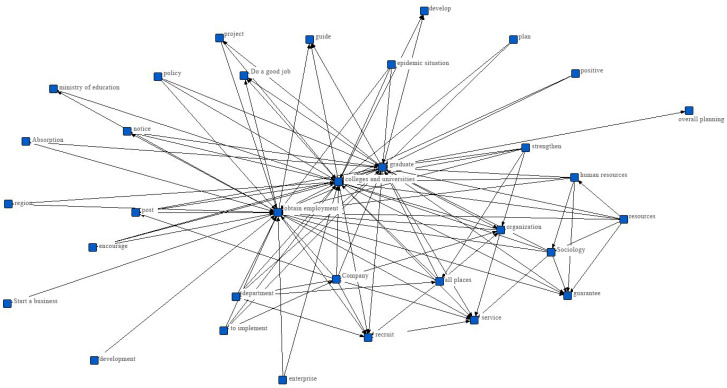
Co-citation networks of keyword in employment promotion policy documents for college graduates.

Based on policy text, this paper uses ROST CM [22,23] software to preprocess the policy text, such as word segmentation and keyword frequency statistics, in order to extract the key content from the policy document. The specific process is as follows: first, the policy text is segmented, then the word frequency of the document after word segmentation is ranked, and finally the word segmentation results are sorted according to the word frequency from high to low. The results are shown in Table 2. In addition, the Ucient software was used to build a co-occurrence network for the documents after word segmentation, and the results are shown in Figure 2. Each node in the network represents a keyword, and if there is a line between nodes, the keywords have a symbiotic relationship. At the same time, nodes are displayed according to the keyword centrality. If the keyword has higher centrality, the keyword frequently appears together with other keywords in the network [24].

It can be seen from the keyword frequency distribution and keyword co-citation networks of the aforementioned policy documents that “employment”, “service”, and “position” rank first among the high-frequency words. This is different from the previous situation for college graduates. The COVID-19 has led universities and companies to cancel offline job fairs for the class of 2020, which are the main job opportunities for fresh graduates. Therefore, during COVID-19, the most important thing for the government is to mobilize all units to implement online employment services, expand employment channels and increase employment opportunities. From the two high-frequency words “entrepreneurship” and “grass-roots level”, we can see that in order to increase the employment opportunities of college graduates, the government has repeatedly mentioned encouraging, supporting and guiding graduates to find jobs at the grass-roots level, stabilizing the environment for innovation and entrepreneurship, and giving full play to the important role of “mass entrepreneurship and innovation” in supporting employment.

### 4.2. Evaluating Employment Promotion Policy Documents for College Graduates Based on PMC Model

At present, the more advanced international policy text evaluation method is the PMC Index Evaluation Model established by Estrada [25]. This model believes that everything is constantly in motion and interconnected, so any relevant variable cannot be ignored. Its innovation is that it uses binarydigits 0 and 1 to balance all variables and emphasizes that the number and weight of variables should not be limited, so that the advantages and disadvantages and internal consistency of a policy can be analyzed from various dimensions [26]. Most existing policy evaluation methods have problems such as strong subjectivity and low accuracy. However, the PMC index model method can largely avoid subjectivity and improve accuracy because it obtains raw data through text mining. In addition, the effectiveness of the PMC model has been verified in the literature [25]. In the policy analysis in this paper, the PMC index model takes variables into extensive consideration, which not only can comprehensively analyze the merits and demerits of a policy, but also has the advantages of index traceability and grade identification, and scientifically quantifies the consistency level of each policy from different dimensions. Therefore, this paper introduces the PMC index model to quantitatively evaluate the employment promotion policy for college graduates under COVID-19 and obtains the key points of the policy content from the outstanding policy documents with a PMC index score of 9–10. Generally, the establishment of a PMC index model includes the following steps: (1) establishing a PMC index evaluation index system, (2) establishing a multi-input-output table, and (3) calculating twolevel variable values and PMC index.

#### 4.2.1. Classifying the Variables and Setting Parameters of PMC Index Model

Referring to Estrada and the existing literatures [27,28,29] and combining with the specific characteristics of college graduates’ employment promotion policy, this paper establishes 10 first-level variables and 66 s-level variables. The results are shown in Table 3.

The weights of the second-level variables in Table 3 are set to the same value, and all the parameter values of the second-level variables are set to binarydigits 0 and 1. If the content of the policy document involves the meaning of the second-level variables, it is assigned the value 1; otherwise, it is 0.

#### 4.2.2. Constructing Input-Output Table

The input-output table is a data analysis framework that can store a large amount of data and use multidimensional measurement of a single variable. It is composed of numerous first-level variables and second-level variables that are not restricted by variables. The first-level variables have no fixed order and are independent of each other, and the weights of the second-level variables are equal [30], as shown in Table 4.

The second-level variables’ values are assigned according to the keywords obtained in Section 4.1. When the policy text data contains the keywords corresponding to the second-level variables, the value is assigned to 1; otherwise, it is 0. Compared with the subjectivity of expert scoring, this method is more objective and scientific.

#### 4.2.3. Calculating PMC Index

The PMC index of the policy documents in Table 1 is calculated below. The calculation method is as follows:

(1) $$X_{i:j}\sim n,$$

*i* is first-level variables; *j* is second-level variables, *i*,*j* = 1,2,3,4,5.…∞.

(2) $$X_{i}=\left(\sum_{j=1}^{n}\frac{X_{i:j}}{n}\right),$$

*n* is the amount of second-level variables, *n* = 1,2,3,4,5.…∞.

(3) $$\begin{array}{l} PMC=X_{1}(\sum_{a=1}^{5}\frac{X_{1:a}}{5})+X_{2}(\sum_{b=1}^{3}\frac{X_{2:a}}{3})+X_{3}(\sum_{c=1}^{5}\frac{X_{3:c}}{5})+X_{4}(\sum_{d=1}^{5}\frac{X_{4:d}}{5})\\ +X_{5}(\sum_{e=1}^{7}\frac{X_{5:e}}{7})+X_{6}(\sum_{f=1}^{9}\frac{X_{6:f}}{5})+X_{7}(\sum_{g=1}^{13}\frac{X_{7:g}}{5})+X_{8}(\sum_{h=1}^{13}\frac{X_{8:h}}{5})\\ +X_{9}(\sum_{k=1}^{5}\frac{X_{9:k}}{5})+X_{10} \end{array}$$

First, determine the value of the second-level variable X*_i:j_* according to Formula (1), then calculate the value of each first-level variable according to Formula (2), and finally bring each first-level variable into Formula (3) to calculate the PMC index of different policies. The PMC index evaluation criteria can be obtained from the literature [21]: 9–10 points (perfect level), 7–8.99 points (excellent level), 5–6.99 points (acceptable level), 0–4.99 points (bad level). This method obtains the ranking and rating of the PMC index of the employment promotion policy for college graduates, and the results are shown in Table 5.

From the evaluation results in Table 5, it can be seen that among the 16 college graduate employment promotion policies, eight of the policy evaluation results are acceptablelevel or above, accounting for 50%, among which two are perfect, and the policy content contained in the perfect policy document is more comprehensive. The target audience is wider, and the steps involved in the implementation measures are more detailed. Therefore, in order to extract the key points in the policy documents, the contents of the P_3_ and P_5_ perfect-level policy documents are selected and summarized. Due to the diversity of the measures proposed in the policy and their different focuses, the content of the policy is divided into four areas: (1) Increase the opportunities for further education and reduce the number of fresh graduates who are in urgent need of employment. (2) Broaden employment information circulation channels, and guide universities and colleges to carry out extensive online employment. (3) Provide employment subsidies, lower employment restrictions, alleviate employment anxiety of recent graduates, and improve employment benefits of recent graduates. (4) Increase position and increase labor demand. According to the above four aspects, the employment promotion policy measures for college graduates can be divided into four categories: channel measures, transference measures, subsidy measures, and position measures.

## 5. Analyzing Social Effects on Employment Promotion Policies for College Graduates

As a reflection of public sentiment and public opinion, online public opinion not only manifests its influence on major developments, but also penetrates into the political level, becoming an important channel for the government to listen to and understand public opinion. In order to dig out the public’s attitude and response to the official employment promotion policy under the COVID-19 pandemic, the corresponding topic comment information on the Weibo is crawled, and social implementation effect of employment promotion policy is analyzed based on the comments.

### 5.1. Acquiring and Preprocessing Data

#### 5.1.1. Acquiring Data

This paper searches related topics for 4 kinds of measures on Weibo and selects the 15 topics discussedmost frequently as the data crawling objects. Each topic is shown in Table 6.

**Figure 3:**
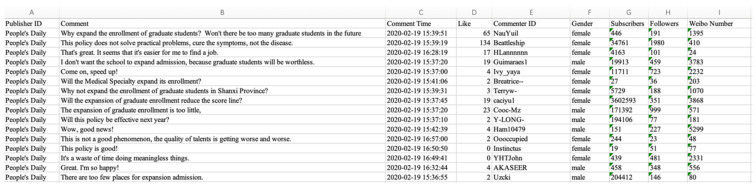
Data format.

This paper trawls the related Weibo comments on 15 topics. The trawled content includes the publisher ID, the content of the comment, comment time, commenter ID, the number of followers, the number of subscribers, and Weibo number. This paper uses python to obtain a total of 65,487 posts, including 9596 posts for channel measures topics, 17,003 posts for transference measures topics, 7671 posts for subsidy measures topics, and 28,849 posts for position measures topics. The data format is shown in Figure 3.

#### 5.1.2. Data Cleaning

Because invalid data and incorrect data inevitably appear in the trawling process, these data are rarely utilized in the analysis process or cause large errors in the results, thusthey need to be deleted. Data cleaning mainly deletes repeatedly collected data, repeated expression words, shorter sentences, meaningless, or unclear sentences. After data cleaning, a total of 61,311 valid posts were obtained.

#### 5.1.3. Word Segmentation and Word Frequency Statistics

As the content of the comments are all in Chinese, the Jieba Chinese word segmentation package [31] is used to perform word segmentation on the Weibo comments in the Python environment and remove stop words that cannot represent text characteristics. Because the research object of this paper is the employment policy for college graduates under COVID-19 pandemic, the nouns that appear frequently in the document after word segmentation are “student, society, employment”, etc., such words are more neutral and have less meaning for word frequency analysis. Therefore, this type of word is also added to the stop word dictionary. On this basis, the top 100 effective high-frequency words are sorted out as follows: “teacher”, “quota”, “postgraduate”, “epidemic”, “fresh graduate”, “fractional line”, “condition”, “file”, “previous graduate”, “full-time”, “employment rate”, “labor force”, “talent”, “young people”, “master”, “quality”, “civil servant”, “part-time”, “housing price”, “Wuhan”, “workload”, “research assistant”, “proportion”, “normal major”, “doctor”, “Guangdong”, “qualification”, “written examination”, “special post teacher”, “Chongqing”, “student source”, “mathematics”, “college promotion”, “unit”, “age”, “college”, “threshold”, “preliminary examination”, “junior college student”, “origin”, “Sichuan”, “energy”, “household registration”, “re-examination”, “unemployment rate”, “poor student”, “welfare”, “enterprise”, “area”, “level”, “accomplishment”, “bachelor”, “doctoral student”, “Shandong”, “Henan”, “junior college”, “registered residence”, “treatment”, “Beijing”, “elementary school”, “salary”, “subsidy”, “university”, “head teacher”, “interview”, “tripartite agreement”, “vocational school”, “contract”, “rural area”, “preschool education”, “ability”, “Anhui”, “township”, “undergraduate”, “Shanghai”, “region”, “city”, “second degree”, “whole country”, “government office”, “hospital”, “institution”, “kindergarten”, “domicile”, “Chinese”, “art”, “engineering”, “nurse”, “pressure”, “agreement”, “experience”, “kindergarten teacher”, “counselor”, “downtown”, “Tianjin”, “other province”, “music”, “English”, “news”, “county town”.

### 5.2. Construction of Evaluation Model for Supporting Policy Measures

An evaluation model for supporting policy measures is constructed here to evaluate and analyze the public support degree of the four types of measures summarized by the above PMC index model to study the social effects of the implementation of the employment promotion policy for college graduates.

#### 5.2.1. Constructing Evaluation Dimension

The degree of support for policy measures needs to be analyzed from multiple dimensions, including the theoretical goals of the policy measures, the people’s expectations of the policy measures, and the specific implementation methods of the policy measures. Most of the previous studies analyzed the policy support degree from one dimension (the theoretical objectives of the policy [32], the expectations of the masses [33,34], the policy means [35], etc.). The coverage of the policy is relatively narrow and lacks objectivity, which affects the scientific statistical results. In order to improve the credibility of the research results, this paper refers to the various evaluation dimensions adopted by the existing research and redefines the evaluation dimensions. Starting from multiple dimensions, it analyzes the degree of public support for various measures of college graduate employment promotion policies. The dimensions are shown in Table 7.

#### 5.2.2. Constructing Comment Topic Identification System

As netizens often evaluate policy measures from different positions and perspectives, each comment may correspond to different evaluation dimensions. This paper uses the framework semantic dictionary matching method, takes the policy review subject word dictionary as the label system, and completes the identification of the corresponding dimensions by extracting and matching the evaluation words of comments. Among them, the policy review topic identification word dictionary is mainly generated based on the frequency of keyword in the comments combined with manual selection. Due to the large number of identified words, the semantic logic induction method is used to summarize and refine it. Sixteen themes are generated: “national condition”, “human resource”, “work treatment”, “work intensity”, “learning form”, “school roll”, “employment agreement”, “employer”, “position”, “examination”, “enrollment”, “region”, “education”, “subject”, “student type”, and “applicable condition”. Combining the evaluation dimension system constructed in Table 7 and the corresponding 16 themes with 4 evaluation dimensions, a comment topic identification system is obtained as shown in Table 8. This can avoid semantic confusion caused by a large number of topic words, thereby improving the data structure and clarifying the evaluation dimension to which the text belongs.

The above-mentioned comment topic identification system is used to map comments to different evaluation dimensions. By identifying and matching comments, a total of 51,567 pieces of comments related to 4 evaluation dimensions were extracted. The specific results are shown in Table 9.

#### 5.2.3. Determining Policy Theme Weight 

TF-IDF weighting method is used to assign weight to each topic here. The TF-IDF method consists of two parts: the TF method and the IDF method. The TF method is to count the keyword frequency. The basic idea is the more times a word appears in the document, the stronger the word is to summarize documents. The IDF method counts how many documents a word appears in. The basic idea is if a word appears in fewer documents, its ability to distinguish between documents is stronger. The calculation formula of the TF-IDF method in this paper is

(4) $$\mathrm{TF}\text{-}\mathrm{IDF}=\frac{n_{i}}{\sum_{k}n_{k}}\times \log\left(\frac{\left|D\right|}{1+\left|D_{i}\right|}\right)$$

where *n_i_* refers to the number of times the topic recognition word *n_i_* under the *i*th topic appears in the review data, ∑*_k_n_k_* is the total number of words in the corpus, and the result of dividing the two is the word frequency. |*D*| refers to the number of comments in the corpus, |*D_i_*| is the number of comments containing the topic identification words under the *i*th topic, and the logarithm is the inverse document frequency. The product of the word frequency and the inverse document frequency is the TF-IDF weight of the *i*th topic. From this, the weight of each theme can be obtained as shown in Table 10.

A theme with a weight greater than 0.1 has a significant impact on policy support degree and is called a key theme. As can be seen from the above table, there are a total of six key themes in the position measures: work treatment, employer, position, enrollment, subject, and student type. This shows that the number of new jobs were created by position measures and that the employment requirements of these new jobs have a greater impact on the public support degree of positional measures. There is a total of four key themes in the transference measures: examination, region, education, and student type. This shows that the specific arrangements for entrance examinations and the object-oriented fairness of the measures have a greater impact on the public support degree of transference measures. There are 4 key themes in the channel measures: human resource, employer, position, and region. This shows that the effectiveness of policy measures in alleviating the employment situation and the fairness of the policy in terms of geographical terms have a greater impact on the degree of support for position measures. There are 5 key themes in the subsidy measures: enrollment, employment agreement, employer, student type, and applicable condition. This shows that the implementation has a greater impact on the degree of support for subsidy measures.

### 5.3. Calculating Theme Emotion Score

Due to the use of sentence structures such as irony in Weibo, the results obtained by the traditional dictionary-based lexical weight accumulation algorithm are not ideal. Because Internet irony often uses some exaggerated rhetoric to express dissatisfaction and irony, such comments often contain strong emotional colors. In order to ensure the accuracy of the emotion score, it is very important to accurately identify and score this type of comments. Therefore, this paper identifies these sentences and revises the emotion score based on punctuation features to improve the accuracy of the results.

#### 5.3.1. Calculation of Initial Emotion Score

The emotion score of a sentence is not only determined by the emotion evaluation word itself, but also affected by degree adverbs, negative words, and punctuation. In order to improve the accuracy of the emotion score calculation, this paper combined the comment and the Chinese grammar dictionary, selected 117 degree adverbs, and defined their respective emotion strengths. The results are shown in Table 11.

Aiming at the negative words appearing in the comments, this paper combines the original negative words in HowNet dictionary and the common phrases in Weibo to sort out a total of 27 negative words after manual screening. In addition to degree adverbs, users often use continuous punctuation (such as “!!!”, “???”) to reflect their own emotions. In this regard, the punctuation at the end of the comment will be identified, and the emotional intensity of various punctuation will be set, as shown in Table 12 below.

In summary, the initial emotional score of the *i*th comment, *E_i_*, is expressed as follows:

(5) $$E_{i}=\sum_{j}^{n}\left[{\left(-1\right)}^{N_{j}}W_{j}P_{m}\prod_{j}^{q}L_{j}\right]$$

where *W_j_* is the *j*th emotion score in comment, and *L_j_* is the emotion strength of degree adverbs before the *j*th emotion word. *N_j_* is the number of negative words before the *j*th emotion word. *P_m_* is the emotion strength of punctuation at the end of the comment. *q* is the amount of degree adverbs before the *j*th emotion word.

#### 5.3.2. Modifying Emotion Score of Irony 

In the Chinese context, irony has various manifestations, and the most common form is rhetorical question. When identifying rhetorical questions, the biggest challenge lies in distinguishing interrogative sentences from rhetorical questions. The common feature of the two is the ending of the question. The difference between the two is that rhetorical questions often contain vocabulary with a certain emotional inclination, while interrogative sentences do not contain emotional inclination. Therefore, the rhetorical question processing rules are as follows:

(6) $$E_{i}^{*}=\{\begin{array}{c} -1\times E_{i}\\ E_{i} \end{array}\begin{array}{c} \mathrm{ending}\;\mathrm{with}\;\mathrm{the}\;\mathrm{question}\;\mathrm{and}\;E_{i}\neq 0\\ \mathrm{other} \end{array}$$

#### 5.3.3. Emotion Score of Various Themes

After the above calculation, the emotion scores of various policy themes are obtained, and the results are shown in Table 13.

It can be seen from Table 13 that the highest emotional score among position measures is “national condition”, with a score of 0.9016, indicating that the public believes that position measures can increase the employment rate of college students and reduce the impact of COVID-19 on the economy. Among position measures, the lowest emotion score is “work intensity” with a score of −1.2313, which shows that the public believes thattreatments of new jobs brought about by position measures needs to be improved. Among the transference measures, the highest emotion score is the “examination” with a score of 1.2514, indicating that the public believes that the transference measures have a positive impact on the entrance examination. Among the transference measures, the lowest emotion score is the “subject” with a score of −1.7181, indicating that the public believes that the subject is not comprehensive enough. Among the channel measures, the highest emotion score is “national condition” with a score of 0.5156, indicating that the public believes that channel measures have a positive impact on increasing the employment rate and promoting the rapid employment of college graduates. Among the channel measures, the region with the lowest emotional score is the “region” with a score of −1.9725, indicating that the public believes that the geographical area targeted by the transference measures is not comprehensive enough. Among the subsidy measures, the highest emotion score is “work treatment” with a score of 2.3995, indicating that the public believes that subsidy measures can effectively improve their own work treatment. Among the subsidy measures, the lowest emotion score is “school roll”, with a score of −3.6528, indicating that the public believes there are some unreasonable aspects in the means related to school roll in subsidy measures.

### 5.4. Analyzing Support Degree for Various Policy Measures

The public support degree for various employment policies is calculated based on the topic weights and emotion scores obtained above. The formula is

(7) $$S=\sum TE^{*}$$

where *S* is the public support degree for the policy measures, *T* is the theme weight, and E^*^ is the theme emotion score. According to the above-mentioned public support degree for various measures, the results are shown in Table 14.

It can be seen from Table 14 that the support for the four types is negative, indicating that the public has a negative attitude towards the employment policy for college graduates under COVID-19. Among them, the degree of support for position measures is close to 0, indicating that the public holds a neutral attitude towards position measures. As the range of support degree for policy measures set in this paper is [−5,5], although the support degree for transference measures is negative, its value is approximately −0.1, which is still in the neutral range. The support degree of subsidy measures and channel measures is lower than −1, indicating that the public is not satisfied with these two types of policies and there is room for further improvement.

Combining Table 10 and Table 13, it can be found that: (1) The emotion scores of “worktreatment”, “employer”, and “position” in the key themes of the position measures are positive, indicating that the public is satisfied with the treatments of newly added positions. However, the emotion score of the “enrollment” is negative and less than −1, indicating that the number of new positions is not satisfactory. In addition, the emotion scores of “subject” and “student type” are less than 0, indicating that the public is dissatisfied with the subject and academic qualifications of graduates for newly added positions. This also reduces the support for position measures, so support for position measures is relatively neutral. (2) For transference measures, under the dimension of “implementation means”, “examination” has the largest proportion and positive emotional score, which makes the support of this dimension positive, indicating that the public is satisfied with the fairness of the public’s specific implementation. However, note that the weight of “target groups” is 0.4743, which is close to 50%, and the support for this dimension is −0.3631, which makes the overall support degree for transference measures not high. In addition, the support degree for the “theoretical objectives” and the “expectations of the masses” is also negative. This is because the people believe that the transference measures are “a temporary solution but not a root cause” and may bring about social problems such as “depreciation of academic qualifications” after implementation. (3) The emotion scores of the four key themes in the channel measures are all negative, and all are less than −1, which is the main reason for the low final support degree of the channel measures. Many people believe that the employment information platform established by channel measures is not well known. Moreover, the policy is mainly for fresh graduates in Hubei: the coverage of the policy is not wide enough. (4) In the evaluation dimension of subsidy measures, the emotion score of “implementation means” is close to −2, and the weight reaches 0.4586. In addition, the emotion score of “target groups” is also lower than −1, which makes the overall support degree for subsidy measures poor. The public believes that the channel measures on student status are not well implemented, and the policies are only targeted at individual regions, thus many regions cannot feel the employment convenience brought by the policy.

## 6. Conclusions

This paper introduced the PMC index model to quantitatively evaluate the employment promotion policy documents of college graduates under COVID-19 pandemic at first. Then, itobtained six acceptable-level policy documents and two excellent-level policy documents, summarizedfour measures according to the key contents of policy documents, as well as constructedsupport evaluation model and analyzed the social effects of the employment promotion policy for college graduates under COVID-19 pandemic.The results showed that the public was generally not satisfied. Among them, the public had a neutral attitude towards position measures and transference measures but was obviously dissatisfied with subsidy measures and channel measures. In this regard, the government should improve and optimize the existing employment policies. Based on the analysis results, this paperput forward the following suggestions:

(1) The public expresses dissatisfaction with the geographical coverage of the four types of measures and the breadth of population coverage. In this regard, the government should optimize, adjust, and expand the subjects and student types covered by the employment policy for college graduates. At the same time, local governments should learn from the advanced experience of other regions to narrow the gap in the implementation of policies among regions, so as to ensure that policies can bring equal benefits to graduates from different regions, schools, and disciplines.
(2) In response to the shortcomings of existing position measures, the government should steadily increase the number of recruits of government agencies and institutions, and can appropriately relax restrictions on the recruitment of subjects and academic qualifications according to the work content and needs of different positions.
(3) The government first needs to pay attention to the problem that transference measures “treat the symptoms but not the root cause” proposed by the public. Second, the government needs to deal with the problem of “difficulties in obtaining employment for college graduates” from a more long-term perspective. It is necessary to foresee that college graduates transferred by transference measures will face employment pressure again in a few years. Therefore, plans should be improved. In addition, in response to the “depreciation of academic qualifications” caused by the implementation of transference measures, the government needs to realize industrial upgrading as soon as possible and create more jobs that require high-end talents to meet the growing demand for high-quality talents in society.
(4) In response to the shortcomings of channel measures, first, the government should increase the promotion of employment information platforms for college graduates and cooperate with social platforms and short video platforms commonly used by young people to increase the popularity of the platform. Second, the government should cooperate with leading companies in various industries to introduce large companies to the platform, and drive many small, medium, and micro-enterprises to settle on the platform. In addition, the government should also cooperate with various universities to guide college graduates to make better use of the platform and increase the utilization rate of the platform.
(5) In response to the shortcomings of subsidy measures, relevant government departments should strengthen supervision and ensure that relevant units and enterprises in various regions implement policies and measures, so that college graduates can truly feel the effect of subsidy measures.

However, this paper still had the following shortcomings, which need further study:

(1) The research object of this paper was the employment promotion policy for college graduates issued by the government from January to July 2020. The impact of the COVID-19 epidemic is still not finished yet, and the government will issue new employment promotion policies. Therefore, further analysis of the effects of the new policy will be carried out in the follow-up.
(2) This paper mainly analyzed the social effects of the implementation of the employment promotion policy for college graduates under the COVID-19 pandemic, so the economic effects of the policy implementation should be analyzed in the follow-up as well.
(3) Based on the data of China’s Weibo [36], this paper evaluated the implementation effect of the employment policy for college graduates issued by the Chinese government. However, COVID-19 has an impact on all countries in the world; therefore, in the future, we will collect data from all countries, conduct targeted research, and give corresponding suggestions.

## Figures and Tables

**Table 1:** Documents of employment promotion policy for college graduates.

No.	Policy Document	Issuing No.	Issuing Department	Issuing Date
P_1_	Notice on Implementing Employment Work during the Period of Epidemic Prevention and Control	Tianjin People’s Office issued (2020) No. 29	Ministry of Human Resources and Social Security of China/Ministry of Education of China/Ministry of Finance of China/Ministry of Transportation of China/National Health Commission	2020.2.5
P_2_	Notice on Carrying out the National Online Joint Recruitment of 2020 College Graduates-24365 Campus Recruitment Service Activities	Ministry of Education of China (2020) No. 2	Office of the Ministry of Education of China	2020.2.28
P_3_	Notice on Carrying out Employment and Entrepreneurship of the 2020 National College Graduates during COVID-19	Ministry of Education of China (2020) No. 2	Ministry of Education of China	2020.3.4
P_4_	Notice on Carrying out Public Recruitment of College Graduates by Public Institutions during COVID-19	Human Resources and Social Security of China (2020) No. 27	General Office of the Organization Department of the CPC Central Committee of China/General Office of the Ministry of Human Resources and Social Security of China	2020.3.11
P_5_	Suggestions on Strengthening and Stabilizing Employment during COVID-19	Office of the State Council of China (2020) No. 6	Office of the State Council of China	2020.3.18
P_6_	Notice on Implementing Some Vocational Qualifications “First Employed then Passed the Exam”	Human Resources and Social Security of China (2020) No. 24	Ministry of Human Resources and Social Security of China/Ministry of Education of China/Ministry of Justice of China/Ministry of Agriculture and Rural Affairs of China/Ministry of Culture and Tourism of China/National Health Commission of China/National Intellectual Property Office of China	2020.4.21
P_7_	Notice on Holding the 2020 National College Graduate Employment Network Alliance Recruitment Week	Ministry of Education of China (2020) No. 7	Ministry of Education of the People’s Republic of China	2020.4.23
P_8_	Notice on Carrying out the Pioneer base for Entrepreneurship and Employment	National Development and Reform Commission of China (2020) No. 310	General Office of the National Development and Reform Commission of China/General Office of the State-owned Assets Supervision and Administration Commission of the Ministry of Education of China/General Office of the Ministry of Human Resources and Social Security of China	2020.4.24
P_9_	Notice on National SME Online Recruitment of College Graduates in 100 Days	Ministry of Industry and Information Technology of China (2020) No. 179	Provincial Department of Industry and Information Technology of China/Provincial Department of Education of China/Provincial Department of Human Resources and Social Security of China	2020.4.27
P_10_	“Notice on Public Recruitment of Kindergarten Teachers in Primary and Secondary Schools in 2020	Human Resources and Social Security of China (2020) No. 28	Ministry of Human Resources and Social Security of China/Ministry of Education of China/Central Planning Office of China/Ministry of Finance of China	2020.5.9
P_11_	Notice on Implementation of the “Three Supports and One Support” Plan for College Graduates in 2020	Human Resources and Social Security of China (2020) No. 57	General Office of the Ministry of Human Resources and Social Security of China/General Office of the Ministry of Finance of China	2020.5.19
P_12_	Notice on Encouraging Scientific Research Projects to Absorb College Graduates	Ministry of Science and Technology of China (2020) No. 132	Ministry of Science and Technology of China/Ministry of Education of China/Ministry of Human Resources and Social Security of China/Ministry of Finance of China/Chinese Academy of Sciences/Natural Science Foundation of China	2020.5.27
P_13_	Notice on Further Development of Research Assistant Positions in Colleges and Universities to Absorb Graduate Employment	Ministry of Education of China (2020) No. 23	Office of the Ministry of Education of China	2020.6.4
P_14_	Notice on Guiding and Encouraging College Graduates to Work and Start Business in Urban and Rural Communities	Human Resources and Social Security of China (2020) No. 53	Organization Department of the Party Committee of each city (prefecture)/Civilization Office of China/Civil Affairs Bureau of China/Education Administrative Department of China/Finance Bureau of China/Human Resources and Social Security Bureau of China/Health and Health Committee of China	2020.6.22
P_15_	Notice on Precise Assistance for Employment of College Graduates from Poor Families in 52 Poverty Counties”	Ministry of Education of China (2020) No. 21	General Office of the Ministry of Education of China/General Office of the Ministry of Human Resources and Social Security of China/General Department of the Poverty Alleviation Office of the State Council of China	2020.7.2
P_16_	Suggestions on Allowing Medical College Graduates to Exempt from Examination to Apply for Practicing Registration of Rural Doctors	National Health Commission of China (2020) No. 11	National Health Commission of China	2020.7.6

**Table 2:** Statistics of keyword frequency in employment promotion policy documents for college graduates.

Keyword	Frequency	Keyword	Frequency
employment	1328	resource	678
graduate	1322	safeguard	676
college	1314	implement	575
recruitment	1166	society	474
service	1130	scientific research	371
company	1120	program	271
entrepreneurship	1111	strengthen	168
position	890	epidemic	166
organization	887	personnel	166
enterprise	882	policy	164
department	781	grassroots	88

**Table 3:** PMC evaluation variables of employment promotion policy for graduates.

First-Level Variables	Second-Level Variables No.	Second-Level Variables Name	Second-Level Variables No.	Second-Level Variables Name
Nature of X_1_ policy	X_1:1_	supervision	X_1:2_	support
	X_1:3_	advisement	X_1:4_	encourage
	X_1:5_	guide		
Time of X_2_ policy	X_2:1_	transition period	X_2:2_	short term
	X_2:3_	this year		
Field of X_3_ policy	X_3:1_	economy	X_3:2_	public management
	X_3:3_	talent	X_3:4_	social security
	X_3:5_	technology	X_3:6_	institution
Function of X_4_ policy	X_4:1_	expand demand	X_4:2_	normative guidance
	X_4:3_	strengthen protection	X_4:4_	institutional constraints
	X_4:5_	optimize system		
Objective of X_5_ policy	X_5:1_	enterprise	X_5:2_	college graduates
	X_5:3_	college	X_5:4_	all provinces, cities, autonomous regions, and municipalities directly under the Central Government
	X_5:5_	directly subordinate agency	X_5:6_	key areas of the epidemic
	X_5:7_	ministries and commissions of the State Council		
Content of X_6_ policy	X_6:1_	resumption of work and production	X_6:2_	employment subsidy
	X_6:3_	employment service	X_6:4_	encourage employment and entrepreneurship
	X_6:5_	stable employment	X_6:6_	broaden employment channels
	X_6:7_	strengthen training	X_6:8_	encourage grassroots work
	X_6:9_	accurate employment assistance		
Issuing agency of X_7_ policy	X_7:1_	Ministry of Human Resources and Social Security	X_7:2_	Ministry of Education
	X_7:3_	Ministry of Finance	X_7:4_	Transportation Department
	X_7:5_	National Health Commission	X_7:6_	provinces and cities
	X_7:7_	Local and subordinate colleges and universities	X_7:8_	General Office of the Central Organization Department
	X_7:9_	Department of Justice (Bureau)	X_7:10_	Department of Agriculture and Rural Affairs (Agriculture, Animal Husbandry and Veterinary Medicine, Fishery) (Bureau, Commission)
	X_7:11_	Department of Culture and Tourism (Bureau)	X_7:12_	Intellectual Property Office (Intellectual Property Management Department)
	X_7:13_	SASAC		
Incentives of X_8_ policy	X_8:1_	employment subsidy	X_8:2_	job creation
	X_8:3_	tax incentives	X_8:4_	talent incentive
	X_8:5_	online employment	X_8:6_	multi-channel employment
	X_8:7_	incentives for primary services	X_8:8_	self-employed
	X_8:9_	skills Training	X_8:10_	employment guidance service
	X_8:11_	encourage teaching	X_8:12_	employment assistance
	X_8:13_	lower the barriers to employment		
Evaluation of X_9_ policy	X_9:1_	clear objective	X_9:2_	feasible plan
	X_9:3_	sufficient reference	X_9:4_	detailed planning
	X_9:5_	encourage employment		
Publication of X_10_ policy	---			

**Table 4:** Input-output table.

First-Level Variables	Second-Level Variables
X_1_	X_1:1_ X_1:2_ X_1:3_ X_1:4_ X_1:5_
X_2_	X_2:1_ X_2:2_ X_2:3_
X_3_	X_3:1_ X_3:2_ X_3:3_ X_3:4_ X_3:5_
X_4_	X_4:1_ X_4:2_ X_4:3_ X_4:4_ X_4:5_
X_5_	X_5:1_ X_5:2_ X_5:3_ X_5:4_ X_5:5_ X_5:6_ X_5:7_
X_6_	X_6:1_ X_6:2_ X_6:3_ X_6:4_ X_6:5_ X_6:6_ X_6:7_ X_6:8_ X_6:9_
X_7_	X_7:1_ X_7:2_ X_7:3_ X_7:4_ X_7:5_ X_7:6_ X_7:7_ X_7:8_ X_7:9_ X_7:10_ X_7:11_ X_7:12_ X_7:13_
X_8_	X_8:1_ X_8:2_ X_8:3_ X_8:4_ X_8:5_ X_8:6_ X_8:7_ X_8:8_ X_8:9_ X_8:10_ X_8:11_ X_8:12_ X_8:13_
X_9_	X_9:1_ X_9:2_ X_9:3_ X_9:4_ X_9:5_
X_10_	---

**Table 5:** PMC index of employment promotion policy documents for college graduates.

	X_1_	X_2_	X_3_	X_4_	X_5_	X_6_	X_7_	X_8_	X_9_	X_10_	PMC Index	Ranking	Depression Index	Rating
P_1_	1	0.33	0.5	0.4	0.71	0.56	0.38	0.23	0.4	1	5.51	5	4.49	acceptable
P_2_	0.4	0.67	0.33	0.4	0.43	0.22	0.23	0.08	1	1	4.76	9	5.24	bad
P_3_	1	0.67	0.67	1	0.43	0.78	0.15	0.69	1	1	7.39	2	2.61	perfect
P_4_	0.8	0.67	0.33	0.6	0.43	0.56	0.23	0.31	0.6	1	5.53	4	4.47	acceptable
P_5_	1	1	1	0.8	0.86	0.89	0.15	0.69	0.6	1	7.99	1	2.01	perfect
P_6_	0.4	0.67	0.5	0.4	0.29	0.22	0.54	0.23	0.8	1	5.05	8	4.95	acceptable
P_7_	0.2	0.33	0.33	0.2	0.43	0.22	0.15	0.15	0.6	1	3.61	15	6.39	bad
P_8_	0.6	0.33	0.33	0.8	0.71	0.44	0.23	0.46	0.8	1	5.7	3	4.3	acceptable
P_9_	0.4	0.33	0.17	0.4	0.29	0.33	0.08	0.08	1	1	4.08	14	5.92	bad
P_10_	0.4	0.33	0.5	0.4	0.29	0.33	0.31	0.23	0.8	1	4.59	11	5.41	bad
P_11_	0.8	0.33	0.5	0.4	0.29	0.33	0.23	0.23	1	1	5.11	7	4.89	acceptable
P_12_	0.6	0.33	0.33	0.4	0.29	0.22	0.31	0.15	1	1	4.63	10	5.37	bad
P_13_	0.6	0.33	0.33	0.4	0.29	0.22	0.31	0.15	0.6	1	4.23	12	5.77	bad
P_14_	0.6	0	1	0.8	0.29	0.22	0.38	0.38	0.6	1	5.27	6	4.73	acceptable
P_15_	0.4	0	0.17	0.4	0.43	0.44	0.23	0.23	0.8	1	4.1	13	5.9	bad
P_16_	0.6	0	0.17	0.4	0.43	0.22	0.08	0.15	0.2	1	3.25	16	6.75	bad
average	0.61	0.39	0.45	0.51	0.43	0.39	0.25	0.28	0.74	1				

**Table 6:** Related Weibo topics.

Policy	Weibo Topic
Channel measures	24.356 all-day online campus employment service
	Encourage multiple methods such as webcasting
	Single assistance between domestic colleges and Hubei colleges
Transference measures	Enrollment of postgraduate students increased by 189,000
	Expand the scale of enrollment for postgraduates and undergraduates
	Expand the postgraduate enrollment of retired soldiers in college
Subsidy measures	Provide employment subsidies for college graduates in many places
	The highest award for innovation and entrepreneurship of Tianjin college graduates is 300,000 RMB
	Find a job within two years and go through the employment procedures according to the current term
	Graduates can keep their household registration files in the school for two years
Position measures	State-owned enterprises expand the enrollment of college graduates this year and next two years
	Expand the recruitment of primary and secondary school teachers
	Implement “first recruited, then passed the exam”
	Special post teachers plan to increase recruitment by 5000
	Develop research assistant position to attract college graduates

**Table 7:** Evaluation dimension.

Evaluation Dimension		Comments
Dimension	Definition	
Theoretical objectives	The theoretical effect to be achieved at the government level under the preset expectations of policy measures	Since the epidemic is so severe this year, it is necessary to introduce policies to ensure employment.
Expectations of the masses	Expected effects of policy measures at the public level	With postgraduate enrollment expansions, the graduate degree will be worthless in the future.
Implementation means	Specific implementation methods and processes of policy measures	The policy was issued too late, the school has already sent the files back.
Target groups	The main body of policy measures	Hope this policy is not just for fresh graduates.

**Table 8:** Comment topic identification system.

Dimension	Theme	Identification Word
Theoretical objectives	National condition	employment rate, unemployment rate, epidemic, housing price
	Human resource	labor force, talent, young people, quality
Expectations of the masses	Work treatment	salary, treatment, subsidy, welfare
	Work intensity	pressure, workload, energy
Implementation means	Learning form	full-time, part-time
	School roll	file, student source, registered residence, origin, household registration, domicile,
	Employment agreement	agreement, tripartite agreement, contract
	Employer	kindergarten, enterprise, university, government office, hospital, institution, vocational school, elementary school, college, unit
	Position	nurse, kindergarten teacher, teacher, civil servant, counselor, head teacher, research assistant, special post teacher
	Examination	written examination, interview, preliminary examination, reexamination
	Enrollment	quota, proportion
Target groups	Region	Shandong, Wuhan, Beijing, rural area, Chongqing, Sichuan, Guangdong, Anhui, Tianjin, area, Shanghai, Henan, whole country, other province, city, downtown, region, county town, township
	Education	bachelor, junior college, Doctor, Master, second degree, college promotion
	Subject	normal major, mathematics, music, Chinese, English, news, art, engineering, preschool education
	Student type	fresh graduate, poor student, doctoral student, postgraduate, undergraduate, junior college student, previous graduate
	Applicable condition	age, qualification, threshold, condition, experience, ability, accomplishment, fractional line, level

**Table 9:** Classification of comments.

Dimension	Theme	Position Measures	Transference Measures	Channel Measures	Subsidy Measures
Theoretical objectives	National condition	2429	728	468	30
	Human resource	1106	871	573	41
Expectations of the masses	Work treatment	3098	388	75	442
	Work intensity	443	435	90	35
Implementation means	Learning form	40	466	37	9
	School roll	160	5	41	1037
	Employment agreement	146	77	327	692
	Employer	2887	522	876	590
	Position	5390	557	795	418
	Examination	1429	1521	43	48
	Enrollment	2890	522	6	380
Target groups	Region	1060	875	1044	376
	Education	786	1876	239	20
	Subject	3179	712	23	73
	Student type	2939	1289	22	675
	Applicable condition	2113	516	38	549

**Table 10:** Weight of policy theme.

Dimension	Theme	Position Measures	Transference Measures	Channel Measures	Subsidy Measures
Theoretical objectives	National condition	0.0675	0.0633	0.0831	0.0078
	Human resource	0.0122	0.0612	0.1001	0.0088
Expectations of the masses	Work treatment	0.1067	0.0304	0.0143	0.0942
	Work intensity	0.0135	0.0513	0.0161	0.0034
Implementation means	Learning form	0.0013	0.0518	0.0081	0.0016
	School roll	0.0081	0.0005	0.0094	0.1889
	Employment agreement	0.0099	0.0024	0.0751	0.1189
	Employer	0.1073	0.0278	0.1853	0.1243
	Position	0.1882	0.0605	0.1767	0.0794
	Examination	0.0528	0.1512	0.0093	0.0079
	Enrollment	0.1005	0.0252	0.0017	0.0565
Target groups	Region	0.0352	0.1010	0.2368	0.0545
	Education	0.0243	0.1478	0.0594	0.0043
	Subject	0.1046	0.0667	0.0062	0.0109
	Student type	0.1061	0.1102	0.0091	0.1277
	Applicable condition	0.0620	0.0486	0.0093	0.1009

**Table 11:** Emotion strength of degree adverbs.

Level	Emotion Strength	Degree Adverb	Amount
High	2	Very, greatly	47
Middle	1.5	too, more	39
Low	0.5	a little	31

**Table 12:** Emotion strength of punctuation.

Punctuation	Emotion Strength
! × *n*(*n* ≥ 1)	1.5 × *n*
? × *n*(*n* ≥ 2)	1.2 × (*n* − 1)
~ × *n*(*n* ≥ 1)	0.8 × *n*

**Table 13:** Emotion score.

Theme	Average Emotion Score			
	Position Measures	Transference Measures	Channel Measures	Subsidy Measures
National condition	0.9016	−0.6212	0.5156	−1.5795
Human resource	0.3536	0.1653	−1.4315	0.1411
Work treatment	0.1158	0.0491	−1.1797	2.3995
Work intensity	−1.2313	−1.3930	−1.025	−1.2938
Learning form	−0.7427	−0.6341	−0.5376	−1.7500
School roll	−0.8052	−1.1915	−0.8409	−3.6528
Employment agreement	0.6784	−0.1235	0.4256	−2.5643
Employer	0.1595	−0.5430	−1.5411	0.5103
Position	0.6182	−0.3463	−1.0509	0.1168
Examination	−0.4551	1.2514	−0.8135	−1.5063
Enrollment	−1.1651	−0.5399	−0.5776	−2.7280
Region	−0.6805	−0.6057	−1.9725	−0.9744
Education	−0.3996	0.7888	0.4036	−1.1675
Subject	−0.2259	−1.7181	0.0531	0.5362
Student type	−0.2467	−0.8378	−0.8663	−0.6121
Applicable condition	0.3725	−0.5268	−1.1908	−1.9126

**Table 14:** Support degree for policy measures.

Dimension	Support Degree for Policy Measures			
	Position Measures	Transference Measures	Channel Measures	Subsidy Measures
Theoretical objectives	0.8178	−0.2047	−0.5484	−0.6674
Expectations of the masses	−0.6851	−0.5797	−1.0978	2.2553
Implementation means	0.0232	0.2851	−0.9883	−1.9147
Target groups	−0.3252	−0.3631	−1.4389	−1.0839
Total score	−0.0018	−0.1373	−1.0557	−1.2809

## Data Availability

The data used to support the findings of this study are available from the corresponding author upon request.
